# The influence of biological sex and sex hormones on bile acid synthesis and cholesterol homeostasis

**DOI:** 10.1186/s13293-019-0265-3

**Published:** 2019-11-27

**Authors:** Taylor Phelps, Erin Snyder, Erin Rodriguez, Hailey Child, Pamela Harvey

**Affiliations:** Department of Molecular, Cellular, and Developmental Biology, University of Colorado at Boulder, Boulder, CO 80309 USA

**Keywords:** Bile, Cholesterol, Nuclear receptors, Estrogen, Hormones, Cytochrome P450

## Abstract

Obesity and elevated serum lipids are associated with a threefold increase in the risk of developing atherosclerosis, a condition that underlies stroke, myocardial infarction, and sudden cardiac death. Strategies that aim to reduce serum cholesterol through modulation of liver enzymes have been successful in decreasing the risk of developing atherosclerosis and reducing mortality. Statins, which inhibit cholesterol biosynthesis in the liver, are considered among the most successful compounds developed for the treatment of cardiovascular disease. However, recent debate surrounding their effectiveness and safety prompts consideration of alternative cholesterol-lowering therapies, including increasing cholesterol catabolism through bile acid (BA) synthesis. Targeting the enzymes that convert cholesterol to BAs represents a promising alternative to other cholesterol-lowering approaches that treat atherosclerosis as well as fatty liver diseases and diabetes mellitus. Compounds that modify the activity of these pathways have been developed; however, there remains a lack of consideration of biological sex. This is necessary in light of strong evidence for sexual dimorphisms not only in the incidence and progression of the diseases they influence but also in the expression and activity of the proteins affected and in the manner in which men and women respond to drugs that modify lipid handling in the liver. A thorough understanding of the enzymes involved in cholesterol catabolism and modulation by biological sex is necessary to maximize their therapeutic potential.

## Background

Bile acids (BAs) are synthesized from cholesterol in liver hepatocytes and are secreted into the small intestine to emulsify and promote absorption of dietary lipids [1]. Approximately 95% of BAs are reabsorbed by the intestinal epithelium and returned to the liver via the portal vein [2]. The remaining 5% of the total BA pool is excreted daily and replaced by hepatic de novo cholesterol synthesis [1, 2]. A small percentage of BAs is not immediately recycled, and these have recently been identified as systemic signaling molecules with important roles in glucose and lipid homeostasis [2].

### Sexual dimorphisms in BA synthesis and excretion

Although many roles have been described for BAs with regard to cholesterol homeostasis and endocrine signaling in both hepatic and extrahepatic tissues, we focus this review on conversion of cholesterol to BAs and sexual dimorphisms in the activity and regulation of enzymes involved in this process. In mice and humans, the rate of BA synthesis and BA pool composition are sexually dimorphic [3]. Wild-type female mice, for example, have a larger total BA pool than male mice; however, females excrete less fecal BA and catabolize less cholesterol via BA production than males [3–5]. Age-related differences in hormone levels are implicated in the differential production of BA in females [6]. Systemic cholesterol homeostasis is achieved by its synthesis and conversion to BAs in the liver as well as feedback mechanisms mediated by BAs. Consideration of sexual dimorphisms in BA synthesis is a critical complement to known modulation of cardiovascular and hepatic diseases by biological sex.

### Synthesis of BAs in the liver

Cytochrome P450s (CYPs) comprise the majority of the estimated 17 enzymes involved in BA synthesis, with abnormalities in their expression or function leading to liver, digestive, and systemic pathologies secondary to elevated cholesterol [1, 7]. CYPs convert 27-carbon (C27) cholesterol to 24-carbon (C24) BAs that are characterized by a carboxylated side chain (carbons 20–24) and hydroxyl groups at various positions on the steroid core (carbons 1–19) (Table 1) [1]. Although two pathways are responsible for their production, 75% of the total BA pool is produced by the classical (neutral) pathway. The production and ratio of the BAs cholic acid (CA) and chenodeoxycholic acid (CDCA), the major BA species in humans, are also mediated by the classical pathway [8]. By contrast, the alternative (acidic) pathway completes side chain oxidation prior to modification of the steroid ring and results in production of CDCA only [9]. The yield of the alternative pathway is significantly smaller than that of the classical pathway and varies between species; in humans, the alternative pathway produces approximately 10% of the total BAs to replace those excreted from the intestines [10]. Nevertheless, the activity of alternative pathway enzymes can be upregulated by excess cholesterol or with hepatic pathology, and using enzymes shared with the classical pathway enzymes, can produce both CA and CDCA [11].

**Table 1:** Major classical and alternative pathway enzymes with subcellular location, reaction type and position of structure modification, and product(s) formed

Major enzymes	Localization	Reaction	Position on Cholesterol	Product
Classical pathway				
CYP7A1	Endoplasmic Reticulum	Sterol ring modification	C7	7a-hydroxycholesterol
AKR1D1	Cytosol	Sterol ring modification	C5	5b-reduced intermediates
CYP8B1	Endoplasmic Reticulum	Sterol ring modification	C12	7a, 12a-dihydroxy-4-cholestan-3-one
CYP27A1	Mitochondria	Side chain modification	C27	27-hydroxycholesterol
CYP3A4	Endoplasmic Reticulum	Sterol ring modification	C4	4alpha-hydroxy-cholesterol
Alternative pathway				
CYP27A1	Mitochondria	Side chain modification	C27	27a-hydroxylcholesterol 25a-hydroxycholesterol 24a-hydroxycholesterol
CYP7B1	Endoplasmic Reticulum	Sterol ring modification	C7	7a-hydroxylated intermediates
AKR1D1	Cytosol			5b-reduced intermediates
CYP27A1	Mitochondria	Side chain modification	C27	CDCA

### Of mice or men

Although mouse models have been critical in identifying the roles of enzymes in the BA synthetic pathways, it is important to note features that distinguish humans from rodents in this regard. Notably, in mice, the yield of the classical pathway represents approximately 60% of the total BAs [12], whereas in humans, this pathway is responsible for 90% of BA synthesis [9]. Additionally, BA species are more variable in mice and include muricholic acids that are not present in healthy humans [13]. Despite these differences, mouse models displaying dysfunction of the classical pathway show significant attenuation in the excretion of fecal BAs similar to that of humans [8]. Where appropriate, we indicate sexual dimorphisms in human and rodent studies throughout the text and conclude with a review of the role of sex hormones in regulation of the genes involved in BA synthesis (Tables 2 and 3). The role and respective sex differences of critical CYPs that participate in the synthesis and metabolism of sex hormones are not considered here but have been reviewed extensively elsewhere [14].

**Table 2:** Sexual dimorphisms in mice lacking enzymes that participate in formation of bile acids with clinical phenotypes in humans (differences between sexes not considered in these studies)

Bile Acid Synthetic Enzyme	Phenotype in knockout in terms of BAs & cholesterol	Human phenotype when gene is mutated
CYP7A1	Lithogenic composition of gallstones with increased dietary cholesterol in females [30]	Statin-resistant hypercholesterolemia [28]
	BA pool - larger in females [30]	
	BA pool composition - higher CA in females [30]	
	Hepatic cholesterol accumulation with increased dietary cholesterol in females (males not reported) [31]	94% reduction in fecal BA excretion [28]
	Maternal consumption of high fat diet results in male offspring with lower expression than females [33]	Premature atherosclerosis [28]
CYP8B1	BA pool increases in male more dramatically than in females [3]	
	Greater compensatory response by CYP7A1 in female knockout, resulting in increased CDCA [3]	
CYP27A1	Sex differences not reported [55, 59]	Cerebrotendinous xanthomatosis [55]
		Vascular and muscle cholesterol deposition [60]
ARK1D1	Higher hepatic BA concentration and lean phenotype in males [69]	
CYP3A4	Sex differences not reported in genome-edited rats [75]

**Table 3:** Regulation of enzymes involved in bile acid synthesis by hormones and their respective hormone receptors

Enzyme	Hormone/receptor	Regulation	Reference
CYP7A1	17alpha-ethynylestrodrial (EE2)/ER-alpha	Downregulates CYP7A1 expression	[123]
	GPR30	Upregulates CYP7A1 expression	[113]
CYP8B1	17alpha-ethynylestrodrial (EE2)/ER-alpha	Downregulates CYP8B1 expression	[123[
	Estrogen (with biliary diversion, not intact enterohepatic circulation)	Downregulates CYP8B1 expression	[124]
CYP27A1	Estrogen/ER-alpha & ER-beta	Downregulates CYP27A1 expression	[122]
	Androgens/androgen receptor	Upregulates CYP27A1 expression	[122]
CYP3A4	Estrogen	Downregulates CYP3A4	[114]
AKR1D1	Testosterone	Inhibits upregulation of AKR1D1 by Estrogen	[114]

## Enzymes of the classical and alternative bile acid synthesis pathways

There are approximately 17 enzymes involved in BA synthesis in the liver. Each is regulated by complex networks that involve both cholesterol and BAs as well as by signaling mediated by sex hormones. We limit our review to highlight those enzymes that (1) have critical roles in BA synthesis that when disrupted lead to clinical pathology in humans and (2) have evidence for regulation by biological sex or sex hormones. Enzymes that lack sexual dimorphisms or regulation by sex hormones are not included in this review but are reviewed elsewhere [2, 7]. For example, although oxysterol 7α-hydroxylase (CYP7B1) is an integral enzyme in the alternative pathway of BA synthesis, disruption of the gene causes no significant pathology in the liver and cholesterol homeostasis is normal. For each enzyme section, we begin with a brief summary of the function of the enzyme and regulation of its expression followed by a description of the phenotype resulting from experimental manipulation of the gene (for complete review see [2, 7]). Each section concludes with a review of the sexual dimorphisms in enzyme expression or function as well as resulting phenotypes.

### CYP7A1

**Fig. 1:**
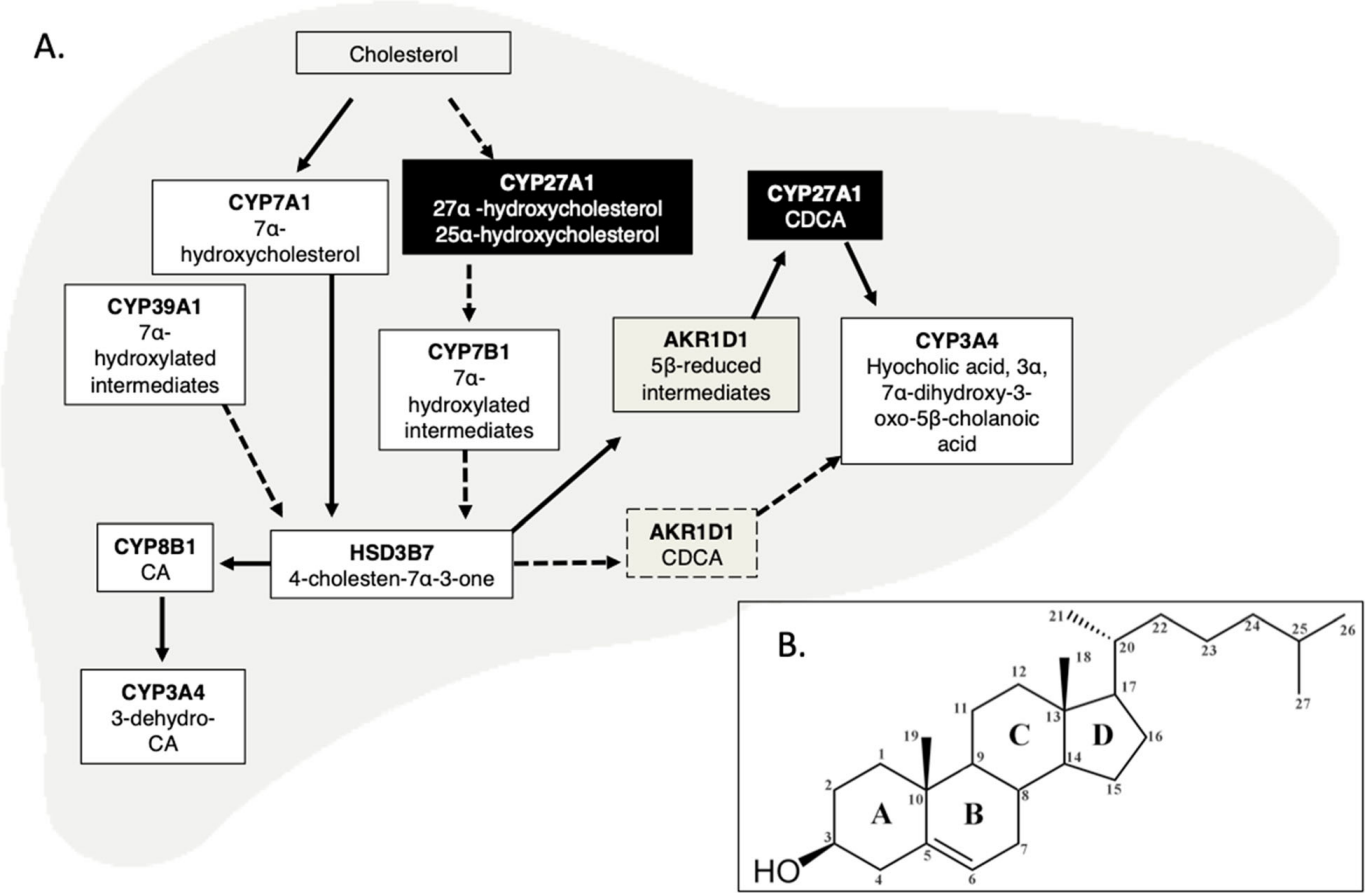
Schematic overview of bile acid synthesis from cholesterol in the liver. Enzymes in white boxes indicate endoplasmic reticulum localization, while gray boxes indicate cytosolic localization. Black boxes indicate mitochondrial membrane localization. Products of enzymatic reactions are listed below each cytochrome P450. Classical pathway enzymes are linked with solid arrows, and alternative pathway enzymes are connected with dashed arrows [16]. Inset: structure of cholesterol with numbered carbons. *CA* cholic acid, *CDCA* chenodeoxycholic acid. Adapted from Fuchs, 2003

The first enzyme in the classical pathway, cholesterol 7α-hydroxylase (CYP7A1), catalyzes the rate-limiting step of BA synthesis from cholesterol [15] (Fig. 1). CYP7A1 hydroxylates the 7α-position on cholesterol to produce 7α-hydroxycholesterol (Table 1). Due to its importance in regulating the rate of BA synthesis, its expression is tightly regulated by a complex mechanism involving cholesterol and BA interactions with nuclear receptors. Transcription is promoted by interactions between cholesterol and the cholesterol-sensor liver X receptor alpha (LXRα) and is negatively regulated by BAs via interaction with farnesoid X receptor (FXR) [17, 18]. BA activation of FXR induces expression of the orphan nuclear receptor small heterodimer partner (SHP), which then interacts with liver receptor homolog-1 (LRH1) to inhibit CYP7A1 expression via its BA response element (BARE) [19, 20]. BAs in the intestines indirectly inhibit CYP7A1 expression by promoting expression of fibroblast growth factor (FGF) 15/19, which is released and binds to hepatic FGF receptor 4 (FGFR4). Subsequent activation of c-Jun N-terminus kinase (JNK) signaling inhibits production of the CYP7A1 transcript [20, 21].

The BA pool in mice lacking *Cyp7a1* is approximately 60% of the size of wild-type mice with a lower concentration of CA [15]. Although BAs are amphipathic molecules, the relationship between the hydrophilic α-side defined primarily by the presence of hydroxyl groups, and of the hydrophobic β-side defined by methyl groups, contributes significantly to the efficiency of sterol solubilization in the intestines [22]. For example, CA and CDCA, both of which are classified as hydrophobic BAs, solubilize sterols at a higher rate in the intestines and regulate expression of genes involved in BA synthesis more efficiently than those that are hydrophilic [23–26]. Reduction in these BAs significantly reduces the uptake of sterols from the intestines; therefore, genetic removal of *Cyp7a1* promotes increased intestinal sterol synthesis and increases 12-alpha-hydroxylase (*Cyp8b1*) expression aimed at restoring appropriate systemic cholesterol concentrations [27]. In agreement with these data, *Cyp7a1* knockout mice are resistant to developing metabolic disorders and have increased glucose sensitivity in response to high dietary fat and cholesterol [15].

As in mice, humans with mutations in *Cyp7a1* that result in loss of function exhibit significantly reduced BA pool as low as 6% of the normal size [28]. *Cyp7a1* mutations correlate with high levels of LDL cholesterol and elevated hepatic cholesterol that can lead to premature atherosclerosis [28]. Supplementation with CA restores BA pool size, BA and sterol excretion, and fractional cholesterol absorption, but also increases intestinal and hepatic cholesterol levels [15, 29]. Additionally, *Cyp7a1* knockout mice expressing human *Cyp7a1* exhibit different responses to altered diets than wild-type counterparts [17]. This discrepancy is likely due to the lack of an LXRα binding sequence in the promoter region of the human gene [17]. BA composition can, therefore, be experimentally manipulated highlighting the potential usefulness of CYP7A1 as a therapeutic target for human disease. Indeed, transgenic mice overexpressing *Cyp7a1* are resistant to obesity, fatty liver disease, and insulin resistance when fed a high-fat diet and exhibit both increased secretion of very low-density lipoproteins (VLDL) and a more hydrophobic BA pool [30].

#### CYP7A1 in males versus females

Male *Cyp7a1* knockout mice have lower levels of CA, smaller BA pools, and lower rates of intestinal cholesterol absorption than females despite the lack of BA synthesis via the classical pathway [27, 30, 31]. Male null mice also demonstrate a negligible response to increased dietary cholesterol. However, females exhibited a threefold increase in hepatic cholesterol levels and a lithogenic gallbladder composition [31, 32]. These data suggest that differences in alternative BA pathway activity in *Cyp7a1*-deficient mice involve sexually dimorphic regulators other than cholesterol [31]. Indeed, recent evidence suggests a sexual dimorphism in the regulation of *Cyp7a1* by thyroid hormone (T3), where T3 can reduce *Cyp7a1* mRNA and protein in male but not female mice that express human CYP7A1 [33]. However, this difference does not seem to extend to humans with thyroid dysfunction [33] (Table 2). Interestingly, maternal diet also affects murine hepatic function in a sexually dimorphic manner; male offspring from mothers who consumed high-fat diets had reduced expression of *Cyp7a1* with concomitant lipid storage in the liver. Male offspring also exhibited increased *Cyp8b1* expression, similar to the *Cyp7a1* knockout mice [34].

### CYP8B1

12-α-Hydroxylase (CYP8B1) catalyzes 12-α-hydroxylation of substrates emerging from both the classical and alternative pathways [35] (Fig. 1). Substrates are either converted by CYP8B1 to precursors of CA, or by aldo-keto redutase 1D1 (AKR1D1) to precursors of CDCA [8]. While CYP7A1 controls the size of the BA pool produced, activity of CYP8B1 controls the ratio of CA to CDCA in the BA pool; decreased activity results in increased CDCA and a more hydrophobic BA pool, whereas increased activity results in increased CA and a more hydrophilic BA pool [36, 37]. Cholesterol uptake, phospholipid transport in the liver, and hepatotoxicity are also regulated by differential expression of CYP8B1 [36, 37]. CA is an established ligand for FXR and association with FXR induces expression of *SHP*, a negative regulator of both *Cyp8b1* and *Cyp7a1* transcription [19]. In the presence of BAs, hepatic nuclear factor 4 alpha (HNF4α) downregulates *Cyp8b1* transcription via upregulation of *Shp* [37]. Peroxisome proliferator activated receptor alpha (PPARα) is also a regulator of both *Cyp7a1* and *Cyp8b1*, and signaling from retinoic acid-related orphan receptor-α may additionally have a role in *Cyp8b1* expression [38, 39]

In *Cyp8b1* knockout mice, the amount of CA produced is significantly reduced [40]. In response, the classical pathway enzyme CYP7A1 is upregulated, due to a lack of negative regulation, to produce more CDCA that compensates for missing CA; the size of the BA pool is not only restored, but is significantly increased in both male and female mice [3, 40]. Knockout of *Cyp8b1* ultimately causes steatorrhea and related symptoms attributed to decreased intestinal absorption of lipids and BA reuptake [35, 40]. However, targeted inhibition protects against development of type 2 diabetes mellitus and cholestasis [35, 40].

Upregulation of *Cyp8b1* when its negative inhibitor SHP is genetically deleted decreases deposition of atherosclerotic plaques when accompanied by apolipoprotien E (APOE) knockout in spite of dietary CA supplementation or high fat [41, 42]. Conversely, genetic removal of both *Cyp8b*1 and APOE increased aortic plaques [43]. Although other regulators have been identified, this apparent susceptibility to the SHP/FXR pathway and reversal by CA makes the gene potentially well suited for targeted pharmacological upregulation [42].

#### Cyp8b1 in males and females

In mice lacking expression of *Cyp8b1*, the total BA pool increases in males by a larger amount than females: 37% in males and 20% in females [3] (Table 2). Moreover, female mice lacking *Cyp8b1* expression also have significantly more CA secondary to higher CYP7A1 activity [3]. As a result of higher BA production in knockout and wild-type females, greater intestinal absorption of sterols is observed compared to males [3]. In fasted wild-type female rabbits, a sevenfold increase in mRNA levels and enzymatic activity was observed; however, in fasted male rabbits, no alteration of either mRNA or activity of CYP8B1 was observed [44, 45].

### CYP27A1

Sterol 27-hydroxylase (CYP27A1) primarily hydroxylates cholesterol to 27-hydroxycholesterol in the first step of BA synthesis in the alternative pathway [46] (Fig. 1). The enzyme also hydroxylates C-27 intermediates produced by CYP7A1 in the classical pathways [47]. Expression and activity of CYP27A1 can be regulated transcriptionally by altering the stability of its mRNA and through variation of available substrate [48, 49]. Specificity protein 1 (SP-1) and HNF4α binding sites and a BARE in the promoter region of the gene have each been reported in human and rat, which when exposed to BAs, produces a downregulation of *Cyp27a1* mRNAs [50, 51]. Like other enzymes in the BA synthesis pathways, upregulation of *Cyp27a1* and BA synthesis is induced by cholesterol [52]. Indeed, overexpression of *Cyp27a1* is sufficient to increase BA synthesis, suggesting a role for the enzyme in responding to hyperlipidemia [52, 53]. In vitro experiments in human hepatocytes have also revealed a role for growth hormone, insulin-like growth factor-1, and glucocorticoids in upregulating activity of CYP27A1 [54]. Further research is necessary to determine the clinical relevance of these data.

More than 30 different mutations in *Cyp27a1* cause cerebrotendinous xanthomatosis (CTX) in humans, which is associated with a variety of symptoms including abnormal synthesis of BAs and deposition of cholesterol and its derivatives primarily in the nervous system and tendons [55]. Patients with CTX lack appropriate regulation of *Cyp7a1*, leading to an accumulation of cholestanol and C-27 bile alcohols [56]. This phenotype manifests as premature, rapidly progressing atherosclerosis and coronary artery disease [57]. However, the symptoms of CTX are not exclusively related to deficiencies in liver BA synthesis, suggesting extrahepatic roles for CYP27A1 or its products in humans. Indeed, *Cyp27a1* is expressed in many extrahepatic tissues, likely due to its presence in macrophages and endothelial cells, where it plays an important role in the hydroxylation of the C-27 vitamin D_3_ [58]. Notably, genetic removal of *Cyp27a1* in mice does not fully recapitulate the symptoms of CTX, revealing an important species divergence in the function or localization of the enzyme [59]. *Cyp27a1* knockout mice exhibit reduced BA synthesis, increased expression of *Cyp7a1*, and elevated serum lipid profiles, similar to patients with CTX [60, 61]. However, none of the tendon or neurological phenotypes are present in the *Cyp27a1* knockout mice [61].

#### CYP27A1 in males and females

Interest in sexually dimorphic drug metabolism and liver disease development lead to several studies describing *Cyp27a1* expression and activity differences in adult males and females. Basal levels of *Cyp27a1* expression appear to be equal in men and women; however, the concentration of its product, 27-hydroxycholesterol, is lower in females suggesting differences in activity levels [62]. The presence of high cholesterol in the diet causes a downregulation of *Cyp27a1* expression in males and females equally [63] (Table 2). The sex of knockout mice was not indicated in previous studies.

### AKR1D1

Aldo-keto redutases are a conserved group of NADPH-dependent oxido-reductase enzymes that reduce ketosteroids [64, 65] (Fig. 1). The 5β-reductases (AKR1D1-3) comprise a unique subfamily that catalyzes the reduction of double bonds of Δ4-3-ketosterols in an efficient and stereospecific manner based on residues presumably located at their active sites [66, 67]. Although the subfamily includes three isoforms, only AKR1D1 is expressed in humans [68].

High concentrations of CDCA are toxic to hepatocytes, and AKR1D1 enzymatic activity is a key regulatory point in controlling the balance of BAs [69]. For example, overexpression of AKR1D1 in isolated human hepatocytes leads to increased expression of CYP3A4 and other CYPs involved in metabolism of xenobiotics. Conversely, genetic reduction of *akr1d1* reduces expression of cytochrome P450s, similar to diabetic patients in whom decreased hepatic expression of *Akr1d1* and decreased production of CDCA are observed [70]. CDCA decreases plasma lipids in hypertriglyceridemic patients; the mechanisms mediating this effect in diabetic patients are currently unknown [71]. Additionally, infants with a deficiency in 5β-reductase activity have reduced primary BA synthesis and accumulation of Δ4-3-keto- and 5α-reduced (allo-) BAs [66]. Effects of this metabolic disorder are severe and manifest as cholestasis and neonatal liver damage, which are likely caused by the accumulation of potentially hepatotoxic levels of BAs [66].

#### *AKR1D1 in males and females*

Male mice with genetic removal of *Akr1d1* exhibit fourfold higher BA concentrations in the liver and lower body fat compared to females [72]. *Akr1d1*-deficient mice also display a sexually dimorphic metabolic phenotype with female mice being protected from the adverse metabolic effects of a high-fat diet. In mature mice lacking *ark1d1*, no differences in glucose tolerance is observed, and mice are the same weight as wild-type counterparts [73]. However, after 20 weeks of high-fat diet feeding, female *Akr1d1* knockout mice are protected from diet-induced weight gain, unlike males, who have enhanced insulin sensitivity, suggesting a role in metabolic diseases [73].

### CYP3A4

Members of the CYP3A family are the most abundant CYP450s in the liver and are responsible for the metabolism of approximately 50% of pharmaceuticals available in the USA [74, 75]. Among the four CYP3A isoforms in humans, CYP3A4 is the most highly expressed; eight CYP3A isoforms are expressed in mice [59]. In addition to its role in drug metabolism, CYP3A4 converts cholesterol to 4β-hydroxycholesterol and regulates lipid metabolism as an activator of the LXRα receptor [75, 76] (Fig. 1). CYP3A4 is also responsible for protecting the liver against the toxic effects of high concentrations of BAs thereby serving as a master regulator of expression of many enzymes involved in BA synthesis to protect against cholestasis [77].

Expression of *Cyp3a* causes an accumulation of 25-hydroxycholesterol, while genetic deletion of the enzyme significantly reduces the concentration [78]. Not only is 25-hydroxycholesterol a precursor to BAs, but it is also an oxysterol that suppresses the sterol sensor SREBP-2 and downregulates de novo cholesterol synthesis [78]. Lower cholesterol levels were observed in livers of *Cyp3a* knockout mice; however, more studies are required to clarify the role of CYP3A4 in cholesterol homeostasis [78]. Differences in CYP3A4 expression between individuals can be greater than 50-fold. While over 30 single-nucleotide polymorphisms have been identified, they occur at a frequency of less than 5% in humans and are consistently heterozygous, suggesting that individual differences may result from other mechanisms [79, 80].

Post-translational modifications are predicted to significantly regulate CYP3A4 activity and expression [80]. The protein has at least three phosphorylation sites, although phosphorylation may be related to ubiquitination [81]. Two miRNAs have also been identified to regulate CYP3A4 [80]. One inhibits expression in human embryonic kidney 293 cells, and the other negatively regulates human pregnane X receptor (PXR) and therefore, indirectly inhibits CYP3A4 translation [80]. In silico methods have identified additional miRNAs that may significantly regulate expression, though more research is needed [80].

#### CYP3A4 in males and females

Significant sex differences attributed to CYP3A4 activity have been described in the context of drug and xenobiotic metabolism. Expression and activity of CYP3A4 are higher in women compared to men, a difference that is diminished after menopause with the loss of estrogen [82]. Differences in rates of cholestasis in women, especially caused by pregnancy, are thought to be attributed to differential regulation of CYP3A4 [83]. However, interpretation of data should be made with caution due to the important role of CYP3A4 in the metabolism of estrogen [84].

## The influence of biological sex on BA synthesis

Biological sex has long been recognized as an important modulator of cardiovascular and hepatic diseases [85–88]. Although differences in body composition, hormonal status, and fat distribution complicate interpretation of data, it is clear that premenopausal women compared to age-matched men are at lower risk of developing non-toxin-related liver and cardiovascular diseases including those attributable to elevated serum cholesterol [89, 90]. The lipid profiles of pre-menopausal women are less pro-atherogenic with higher concentrations of high-density lipoproteins containing cholesterol [91]. Sexual dimorphisms in serum cholesterol extend to the liver where sex differences are also observed in BA pool composition and size [92, 93].

The release pattern of growth hormone and subsequent control of signal transducer and activator of transcription 5b (STAT5b) are involved in sexual dimorphism of hepatic CYPs [94]. Other hepatic transcription factors involved in sex-specific expression of P450s include hepatocyte nuclear factor 4α (HNF4α) [69] and retinoid X receptor (RXRα), the co-receptor for many nuclear receptors in hepatocytes [95]. *When HNF4*α *expression is removed, for example,* 372 sex-specific genes are specifically affected in the livers of male mice versus only 61 in the females. Additionally, in female mice, the BA pool is approximately 60% larger and more hydrophobic than in males, and higher fecal levels of excreted BAs are also observed in females [91, 93]. The composition of the bile acid pool is also sexually dimorphic with females producing more CDCA than males [96]. The excess BAs cannot be attributed to *Cyp7a1* expression, which is paradoxically lower in females [4]. However, when challenged with a high-cholesterol diet, female mice exert a 50% higher rate of CYP7A1 activity compared to males [4]. These data significantly confound interpretation of data from rodent models: unlike female mice, women have lower BA pool size compared to men [92] (Table 2).

## The role of sex hormones in cholesterol homeostasis

Female sex and estrogens are emerging as important regulators of BA production and through critical hepatic feedback mechanisms, serum cholesterol levels. Most of the early data on sex differences in serum lipid profiles, BA synthesis, and BA pool composition were derived from observations of pre- versus postmenopausal women, of individuals receiving estrogen supplementation, and of women with polycystic ovarian syndrome in whom circulating sex hormone levels are abnormal [97, 98]. Nearly half of women administered an estrogen receptor (ER) antagonist as a treatment for certain breast cancers develop hepatic steatosis within 2 years of beginning treatment [99]. Similarly, mice that are deficient in estrogen or are not responsive to estrogen signaling are obese and have elevated triglycerides levels that are linked to the development of hepatic steatosis, a condition that is reversible through administration of estrogen [100, 101]. Steatosis that develops in the setting of depleted estrogen may be further exacerbated by exposure to endocrine disruptors with possible estrogen-blocking effects like bisphenol A present in many plastics [102]. Interestingly, in women, high levels of circulating estrogen during pregnancy are associated with development of cholestasis via a mechanism that likely involves inhibition of BA transport to the liver from the intestines rather than synthesis [103, 104]. These conflicting effects may also be explained by the use of both physiological and non-physiological concentrations of estrogen. Despite this, both synthetic and endogenous estrogens have been implicated in sex differences observed in liver dysfunction and are thought to be generally beneficial at normal levels in premenopausal women in terms of preventing and limiting progression of liver and cardiac diseases [105, 106].

### Transcriptional effects of estrogen

Estrogen exerts genomic effects through ligand-bound ERs that translocate to the nucleus and bind to estrogen response elements (EREs). Non-genomic effects are also mediated by membrane-bound ERs via activation of cell signaling cascades [107]. Although the impacts are best described in breast cancer cells, the significance of the modulatory effects of estrogen in the liver is being explored. Estrogen-induced cholestasis is caused by reduced BA synthesis and transport [108]. Hepatocytes express ERα and are, therefore, responsive to both the genomic and non-genomic effects of estrogen [109]. Rat hepatocytes exposed to physiological levels of estrogen exhibit increased CYP7A1 activity along with small transient increases in BA production [110]. However, in vivo effects appear to be diet- and time-dependent. Similarly, livers of ovariectomized baboons on a high fat and cholesterol diet exhibited higher activity of CYP7A1 [111]. Single injection of supraphysiological concentrations of estrogen in rats did not induce changes in CYP7A1 activity at the level of the microsome, whereas 21-day treatment inhibited activity [112]. It is, therefore, unclear whether cholestasis caused by various estrogen supplementation therapies is due solely to altered CYP7A1 expression or activity [113, 114]. Through activation of the estrogen receptor ER-α, synthetic estrogen also upregulates *Cyp7b1* and decreases CYP8B1 signaling; ER-β does not appear to have a role in regulation of BA synthesis enzymes. A recently identified but not well understood estrogen receptor, GPR30, also appears to positively regulate expression of *Cyp7a1* [115] (Table 3).

An ERE has been identified in the promoter region of *Akr1d1* [64]. Higher levels of estrogen may help to reduce the effects of AKR1D1-deficient individuals, since testosterone is an inhibitory substrate for AKR1D1 [116]. Testosterone has two binding sites that both block binding of other hormones like estrogen to AKR1D1 [116]. EREs have not been identified in many cytochrome P450s involved in BA synthesis; however, other proteins and sequences like the transcription factors activator protein 1 (AP-1) and Sp-1 and half-palindromic estrogen response sequences (half-sites) in promoters can mediate the transcriptional activity of nuclear ERs. Estrogen interacts with Sp-1, and this association is required for enhanced transcription of many genes including *RXRα* and *LXRα* [117]. In agreement with this, expression of *RXRα* is significantly higher in the livers of females compared to males [118]. Additionally, CYP3A4 has an important role in the 4-hydroxylation of estrogen, the first step in hepatic metabolism of estrogens [84]. Accordingly, estrogen itself negatively regulates expression of *Cyp3a4*, likely through interactions with ERs in the promoter region of the gene [119, 120]. Indeed, as age increases and estrogen levels decline in women, hepatic levels of CYP3A4 are reduced [119].

Estrogen exerts effects on BA synthesis that influence enzymatic activity as well as BA pool composition. *Cyp27a1* expression, for example, is inhibited in HepG2 liver-derived cells by estrogen treatment. Both ERα and ERβ associate with the promoter region of the *Cyp27a1* gene to inhibit expression; conversely, androgens promote expression of *Cyp27a1* in the same cell line likely via induction of JNK signaling [121, 122]. Interestingly, CYP27A1 enhances ER-ERE interactions in liver cells and may promote upregulation of *Cyp7b1* [123]. Transfection of human embryonic kidney 293 cells with ERα and ERβ combined with estrogen treatment upregulates expression and activity of CYP7B1 [124]. *Cyp7b1* expression is negatively regulated by androgens in prostate cancer cells, in which opposing effects of estrogen are observed [124]. In agreement with these data, examination of the composition of the BA pool has revealed that men have higher CDCA than women [6]. Additionally, in animal models, the concentration of CDCA in bile is reduced with estrogen signaling through ERα [125, 126].

## Conclusions

Drug development for atherosclerosis and BA synthesis deficiencies are increasingly focused on BA biosynthetic pathways. Potential pharmacological targets include the nuclear receptors FXR and SHP; however, excretion of BAs to prevent cytotoxic concentrations will need to be considered. For example, although SHP itself lacks a DNA-binding domain, it interacts with multiple nuclear receptors including ERs, thereby inhibiting their transcription [127, 128]. The SHP promoter harbors an AP-1 binding site that when mutated, removes negative regulation induced by BAs [129]. The biological effects of estrogen on these nuclear receptors appear to vary based on cell type; therefore, it is critical to thoroughly examine their effects in hepatocytes and in the liver in vivo.

Sex differences in the therapeutic response to compounds that target BA synthesis may vary significantly not only with respect to the CYPs that mediate drug metabolism but also in the CYPs that regulate BA concentration and composition between the sexes. Lessons from cardiovascular disease should inform the development of these therapies, and a complete understanding of molecular sexual dimorphisms regulating BA synthesis will help in addressing these issues. Importantly, post-menopausal women may be less sensitive to drugs that intend to increase BA synthesis due to diminished levels of estrogen that normally promote activity of enzymes that produce BAs. Whole-genome examination of promoters for canonical ERE s has been performed, revealing no perfect or near-perfect estrogen binding sites for enzymes involved in BA synthesis except AKR1D1 [130]. However, half-ERE sites that bind SP-1 and AP-1 sites should be considered in greater detail to understand the role of estrogen in regulation in BA synthesis and cholesterol homeostasis in the liver.

## Perspectives and significance

Cholesterol homeostasis has been recognized as an important modulator of the cardiovascular system in health and disease. Indeed, drugs that lower systemic cholesterol such as statins that reduce hepatic cholesterol production improve outcomes of cardiovascular diseases. However, neglecting to consider sex differences in the expression and activity of lipid-handling proteins targeted by cholesterol-lowering drugs has led to limitations in their utility. For example, women experience a significantly higher incidence of myalgia and reduced survival benefit from statins compared to men (Legato et al., [131]). Strategies that reduce cholesterol through modulation of BA synthesis may benefit those who cannot tolerate statins or for whom the drugs are ineffective. To avoid unexpected effects due to biological sex, development of these drugs should address sex differences in the enzymes that produce BAs.

Sex differences in BA synthesis have been reported in humans and rodents for nearly 50 years. Lessons from studies on cholesterol in cardiac health should inform further examination of the roles of both estrogen and androgens in regulating expression of enzymes involved in BA synthesis. We recommend systematic experiments that include addition of exogenous estrogen and androgens and gonadectomized males. This system would allow examination of the roles of both androgens and estrogen. The resulting phenotypes could reveal important information about not only mechanisms regulating BA production but also about the possible hepatic effects of gender-affirming hormone supplementation in transgender individuals, a vastly underrepresented field of study. Comparable studies performed in mice that examine cardiac function have found detrimental effects in gonadectomized mice receiving doses of estrogen relevant to the serum of pre-menopausal females. The same may be true for BA synthesis and cholesterol homeostasis because similar mechanisms mediate the regulation of genes important to bile acid synthesis.

## Data Availability

Not applicable.

## Abbreviations

AKR1D1: Aldo-keto reductase 1D1
AP-1: Activator protein 1
APOE: Apolipoprotein E
BA: Bile acid
BARE: Bile acid response element
CA: Cholic acid
CDCA: Chenodeoxycholic acid
CTX: Cerebrotendinous xanthomatosis
CYP: Cytochrome P450
CYP7A1: Cholesterol 7α-hydroxylase
CYP7B1: Oxysterol 7α-hydroxylase
CYP8B1: 12-Alpha-Hydroxylase
CYP27A1: Sterol 27-hydroxylase
ER: Estrogen receptor
ERE: Estrogen response element
FGF: Fibroblast growth factor
FXR: Farnesoid X receptor
HNF4α: Hepatic nuclear factor 4 alpha
JNK: c-jun N-terminal kinase
LRH1: Liver receptor homolog 1
LXRα: Liver X receptor alpha
PXR: Pregnane X receptor
RXRα: Retinoid X receptor alpha
SHP: Small heterodimer partner
SP-1: Specificity protein 1
SREBP: Sterol regulatory element-binding protein
STAT: Signal transducer and activator of transcription
